# What Is a Solitary Keratoacanthoma? A Benign Follicular Neoplasm, Frequently Associated with Squamous Cell Carcinoma

**DOI:** 10.3390/diagnostics11101848

**Published:** 2021-10-07

**Authors:** Azusa Ogita, Shin-ichi Ansai

**Affiliations:** Division of Dermatology and Dermatopathology, Nippon Medical School Musashi Kosugi Hospital, Kawasaki 211-8533, Japan; azu@nms.ac.jp

**Keywords:** keratoacanthoma, squamous cell carcinoma (SCC), keratoacanthoma-like SCC, keratoacanthoma with malignant transformation, crateriform neoplasms, crateriform verruca, crateriform seborrheic keratosis, crateriform Bowen disease, crateriform SCC arising from actinic keratosis, crater form of infundibular SCC

## Abstract

We present histopathological criteria for diagnosing keratoacanthoma (KA). In KA, four histological stages are recognized, which are the early/proliferative stage, well-developed stage, regressing stage and regressed stage. In diagnosing KA, we emphasize that KA consists of the proliferation of enlarged pale pink cells with ground glass-like cytoplasm without nuclear atypia, other than crateriform architecture. KA sometimes exhibits malignant transformation within the lesions. We describe the characteristics of benign and malignant epithelial crateriform tumors that should be differentiated from KA. We also present the data of histopathological diagnosis of lesions clinically diagnosed as KA, its natural course and related lesions after partial biopsy, and incidence of crateriform epithelial neoplasms. Based on these data, we recommend complete excision of the lesion when KA is clinically suspected, especially when the lesion is located on a sun-exposed area of an elderly patient. If complete excision is impossible, partial excision of a sufficient specimen with intact architecture is required. In such a case, however, careful investigation after biopsy will be needed, even if the histopathological diagnosis is KA, because there is some possibility that a conventional SCC lesion remains in the residual tissue.

## 1. Introduction

Keratoacanthoma (KA) often occurs in a solitary form and exhibits a distinct clinical and histopathological presentation [1]. Whether KA is benign or malignant, i.e., squamous cell carcinoma (SCC) that is one of the most common malignant tumors affecting the akin and of which characteristic is the abnormal and quick growth of keratinocytes in the epidermis, often secondary to ultraviolet or sunlight exposure [2], or not, has been a controversial issue for many years, although there have been many studies concerning the differentiation of KA and SCC [3]. Such confusion is mainly based on similarity of histopathological findings between KA and SCC and lack of accepted reliable histopathological criteria in diagnosing KA [4]. Furthermore, few cases of KA exhibit distant metastasis and tumor-related death [5,6]. Therefore, KA was classified into low-grade SCC in the recent WHO classification of cutaneous tumors [1]. On the other hand, Misago and colleagues suggested that KA is either a benign lesion or a distinct borderline malignant entity that is fundamentally different from conventional SCC and features follicular (infundibular/isthmic) differentiation characterized by the involvement of continuous multi-follicular infundibula [7,8,9,10,11,12]. They also emphasized that KA consists of the proliferation of enlarged pale pink cells with ground glass-like cytoplasm without nuclear atypia, at least in a part of the lesion, and it relatively frequently exhibits malignant transformation. We think that this opinion explains most phenomena about the relationship between KA and SCC. From such points of view, we consider the discussion concerning whether KA is SCC to be meaningless. We are convinced that KA is a benign epithelial neoplasm with follicular differentiation that sometimes grows conventional SCC within the lesion.

In this article, we want to present the true characteristics of solitary KA based on its distinctive histopathological criteria, in addition to histopathological findings of other epithelial crateriform tumors that should be differentiated from KA. Our classification of epithelial crateriform tumors is stated in Table 1.

## 2. Clinical and Histopathological Characteristics of Solitary KA

### 2.1. Clinical Findings

Solitary KA usually develops on sun-exposed areas of elderly patients. Its clinical findings are characterized by a flesh to pink colored crater-like nodule with a central keratotic plug. An essential clinical characteristic of solitary KA is its self-limiting course, with rapid enlargement within several weeks and spontaneous regression within several months. Such a clinical course is highly important in diagnosing KA.

### 2.2. Histopathological Findings

#### 2.2.1. Histopathological Stages

Solitary KA has different histopathological features depending on the stage of the lesion at the time of biopsy or resection [7,12,13]. Four histological stages of KA are recognized, which are the early/proliferative stage, well-developed stage, regressing stage and regressed stage. It is highly important that excisional biopsy or partial biopsy including the center and both sides of KA be performed for correct histopathological diagnosis.

#### 2.2.2. Mutual Findings among Stages

**Figure 1:**
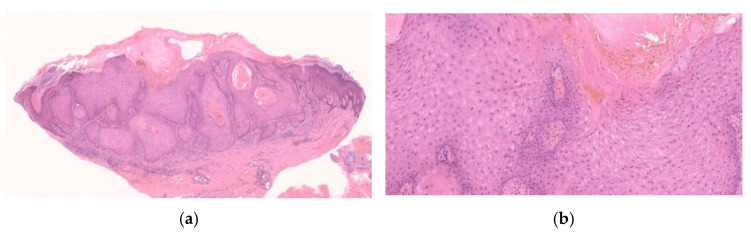
Histopathological findings of KA en plaque/nodule. Gross findings of the lesion reveal an exo-endophytic and non-crateriform architecture (**a**). The lesion consisted of proliferation of large pale eosinophilic cells with a few layers of basophilic cells at their periphery (**b**). Large pale eosinophilic cells show no nuclear atypia (**b**).

KA histopathologically exhibits characteristic findings through all stages except in the regressed stage. These include an exo-endophytic architecture, a relatively well-defined, almost symmetrical outline and a multilobular lesion with a central keratinous plug. It also presents overhanging epithelial lips covered with normal epidermis. Furthermore, other findings should be emphasized: (i) presence of invaginated infundibular structures (laminated keratinization) and lobules with enlarged pale pink cells with ground glass-like cytoplasm, which generally lack nuclear atypia; (ii) lobules of large pale eosinophilic cells with a few layers of basophilic cells at their periphery; (iii) possible nuclear atypia or mitotic figures, limited to the peripheral areas of the basophilic cells; and (iv) minimally infiltrating borders. In particular, proliferation of enlarged pale pink cells with ground glass-like cytoplasm without nuclear atypia is the most important finding in diagnosing KA and differentiating KA from SCC. In KA, the crateriform architecture is characteristic and can be recognized in most cases, but that is not essential. We previously reported cases having the same components as conventional KA without the crateriform architecture as keratoacanthoma en plaque/nodule [14] (Figure 1).

#### 2.2.3. Early/Proliferative Stage

**Figure 2:**
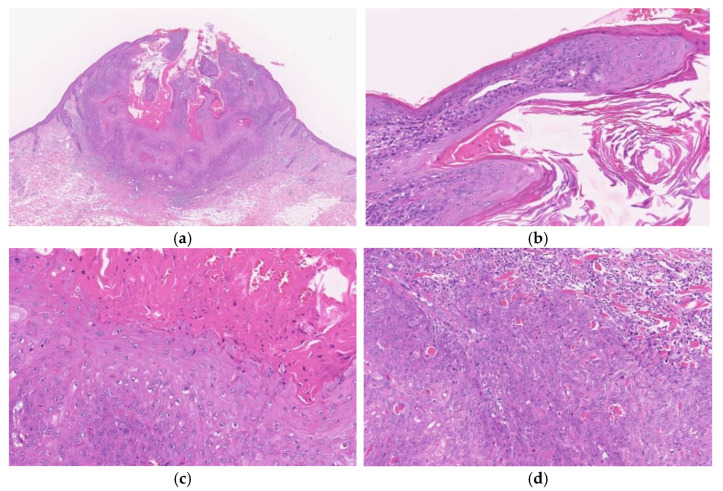
Histopathological findings of KA at the early/ proliferative stage. Gross findings of the lesion include crateriform architecture with a central keratinous plug (**a**). A lip-like structure is observed (**b**). Pale pink keratinocytes with a glassy appearance are observed. In the deeper areas, pale pink keratinocytes with a glassy appearance are noted (**c**). The deeper areas of the lesion are poorly demarcated from the surrounding stroma and exhibit slightly invasive growth (**d**).

The early/ proliferative stage of KA is histopathologically characterized by several keratin-filled invaginations of the epidermis or infundibulum, demonstrating a laminated pattern of keratinization, often with prominent keratohyalin granules. In the deeper areas, pale pink keratinocytes with a glassy appearance are observed. The deeper areas of the lesion are sometimes poorly demarcated from the surrounding stroma and exhibit slightly invasive growth (Figure 2).

#### 2.2.4. Well-Developed Stage

**Figure 3:**
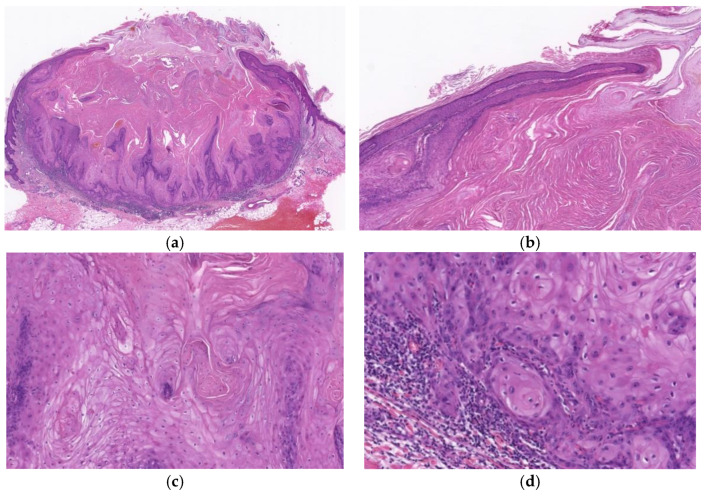
Histopathological findings of KA at the well-developed stage. Gross findings of the lesion include crateriform and exo-endophytic architecture with a central keratinous plug (**a**). Overhanging epithelial lips with a normal overlying epidermis are observed (**b**). Characteristic proliferation of large pale pink cells with a glassy appearance showing compact keratinization is observed in most parts of the lesion (**c**). The deeper areas of the lesion are slightly poorly demarcated from the surrounding stroma and exhibit slightly invasive growth (**d**).

The well-developed stage of KA exhibits the following histopathological findings: (i) characteristic symmetric, crateriform, exo-endophytic architecture; (ii) contiguous, dilated infundibular structures (multilocular and multilobular) with a central large keratotic horn situated above isthmic differentiation; (iii) overhanging epithelial lips with a normal overlying epidermis; and (iv) characteristic neoplastic lobules with isthmic differentiation (proliferation of large pale pink cells with a glassy appearance demonstrating compact keratinization) in most parts (Figure 3). There are also sometimes fine keratohyalin granules or focal parakeratosis.

#### 2.2.5. Regressing Stage

**Figure 4:**
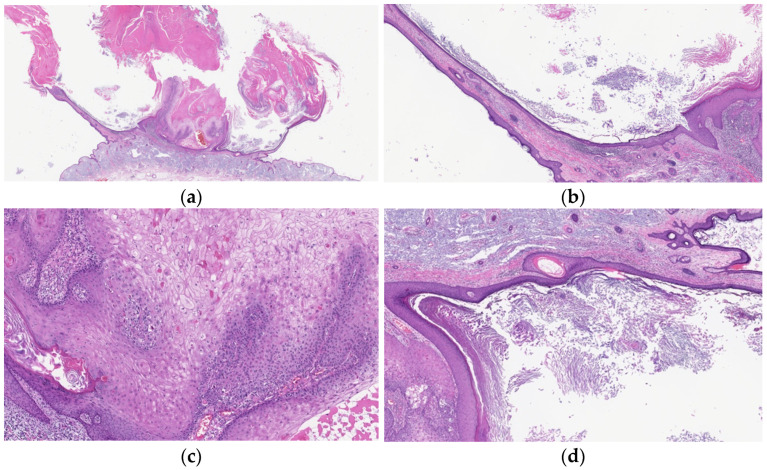
Histopathological findings of KA at the regressing stage. Gross findings of the lesion include crateriform architecture (**a**) and a lip-like structure (**b**). The lesion shows infundibular characteristics of laminated keratinization (**b**,**d**) and often loses the pale pink keratinocytes with a glassy appearance (**c**). Fibrosis in the dermal papillae and mixed cell inflammation are also observed (**b**).

The regressing stage of KA maintains a crateriform architecture, but it becomes one or two keratin-filled and shallow crateriform structures (Figure 4). The regressing stage KA again exhibits infundibular characteristics of laminated keratinization and the pale pink keratinocytes with a glassy appearance are often lost. Fibrosis in the dermal papillae and mixed cell inflammation are also noted (Figure 4).

#### 2.2.6. Regressed Stage

**Figure 5:**
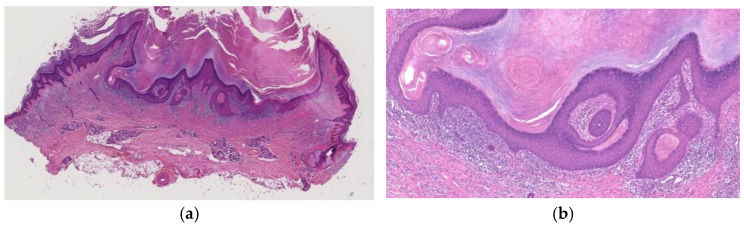
Histopathological findings of KA at the regressed stage. A depressed epidermal lesion with overhanging and rising edges is observed (**a**), and the epidermis is flattened and atrophic with loss of rete ridges (**b**).

The regressed stage of KA is a depressed epidermal lesion with overhanging or rising edges, and the epidermis is flattened and atrophic with loss of rete ridges (Figure 5).

## 3. Diagnostically Problematic Lesions, KA with a Conventional SCC Component (KASCC)

**Figure 6:**
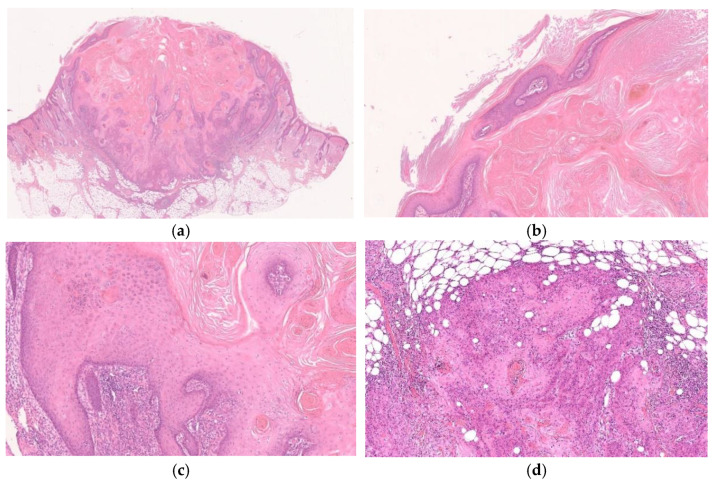
Histopathological findings of KA-like SCC. Gross findings of the lesion include crateriform architecture with a central keratinous plug (**a**). In part of the lesion, the histopathological features of KA are observed (**b**,**c**), whereas the SCC component is composed of tumor cells with keratinocytic differentiation and apparent nuclear atypia and shows invasive growth pattern (**d**).

**Figure 7:**
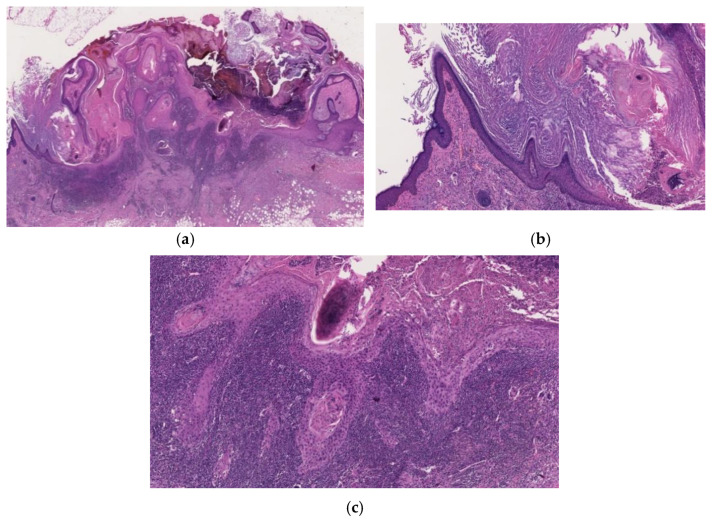
Histopathological findings of mKA. Crateriform architecture is observed, and there is a clear distinction between KA and SCC (**a**). Both sides of the lesion show regressing KA (**b**), whereas the SCC component is in the center (**c**). The boundary between the two components is clear-cut (**a**).

KA-like SCC [9] and KA with malignant transformation (mKA) [15,16] are types of KASCC [10,12,13]. Both types have a component with the histopathological features of KA, e.g., an exo-endophytic lesion formed by invaginated infundibulum and lobules with large pale pink cells having a glassy appearance, generally without nuclear atypia. In KA-like SCC, conventional KA components and SCC components are relatively ill-demarcated and often admixed (Figure 6 and Figure 7). On the other hand, mKA exhibits a well-demarcated contrast between typical KA and SCC sections (nests of anaplastic cells of different shapes and sizes). However, differentiation between these two conditions is often difficult and we found no difference between them in clinical course; therefore, we recommend these tumors be unified as KA with a conventional SCC component (KASCC). KA has a somewhat asymmetrical outline and focally prominent infiltrating border. KA-like SCC is also diagnosed when nuclear atypia is observed in most of the cells constituting the KA-like component (invaginated infundibular structures and lobules of large, pale pink cells). The KA components may be in any stage: early/proliferative, well-developed or regressing. Ratios of the KA and SCC components can vary in each lesion or even in different sections of a single lesion [9,12].

## 4. Other Crateriform Tumors

### 4.1. Benign Neoplasms

#### 4.1.1. Crateriform Verruca (CFV)

We previously reported crateriform epithelial tumors exhibiting some histopathological overlap with KA that failed to meet all of the histological criteria for a diagnosis of KA and had some verrucous features, and we proposed the term crateriform verruca (CFV) to differentiate these verrucous neoplasms from KA [17]. CFV often is diagnosed as KA or verruca. Compared clinically with KA, CFV is smaller despite its longer duration. The common sites of CFV are sun-exposed areas, especially the face and neck [17].

**Figure 8:**
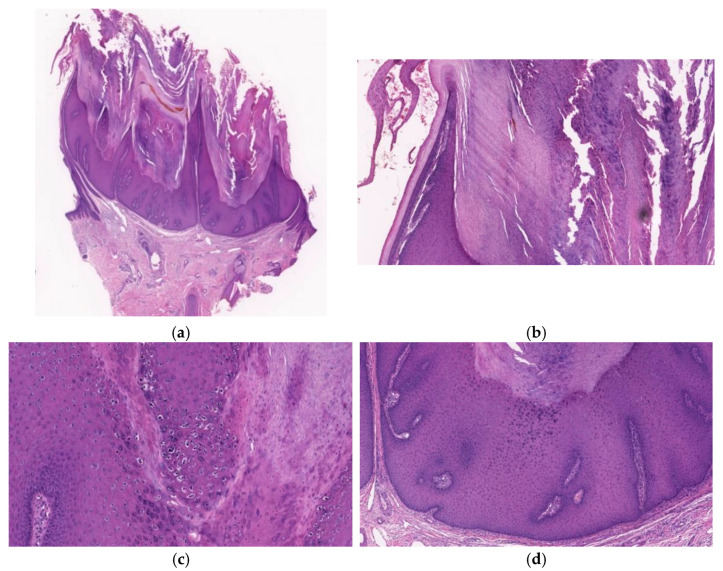
Histopathological findings of CFV. A crateriform configuration with finger-like exophytic projections accompanied (**a**) by epithelial lip-like structures at the periphery (**b**) is exhibited. Arborization is observed and the base is well demarcated without endophytic growth (**a**). Focal hypergranulosis and koilocytes are visible between the papillary projections (**c**). The lesion consists of several lobular structures composed of the proliferation of keratinocytes of a similar size and regular arrangement (**d**).

Histopathologically, CFV is characterized by finger-like exophytic projections associated with hyperkeratosis (parakeratosis or orthokeratosis), focal hypergranulosis (koilocytes are not always prominent) and acanthosis, together with epithelial lip-like structures at the periphery (Figure 8). Characteristic inturning of elongated rete ridges (arborization) is usually observed (Figure 8). CFV can be differentiated from KA because it consists of several lobular structures composed of the proliferation of keratinocytes of a similar size and regular arrangement and because the base of CFV is well demarcated without endophytic growth. Cells with a large eosinophilic cytoplasm may be found in some parts of CFV, but these cells exhibit no downward proliferation unlike in KA (Figure 8). There is generally no nuclear atypia in the basal cell layer, or it is very mild if present, in contrast to the obvious nuclear atypia and mitosis of proliferating keratinocytes at the periphery of early-stage KA. There is either no inflammatory cell infiltration or slight infiltration (mainly lymphocytes and plasma cells), whereas KA in the regressing stage demonstrates fibrosis of dermal papillae and mixed inflammatory cell infiltration. CFV with large pink cytoplasm and trichilemmal keratinization is also histopathologically similar to so-called trichilemmal keratosis (horn) [18,19], the main histological features of which are trichilemmal keratinization and verrucous epidermal hyperplasia composed of large pale staining keratinocytes.

#### 4.1.2. Crateriform Seborrheic Keratosis (CSK)

**Figure 9:**
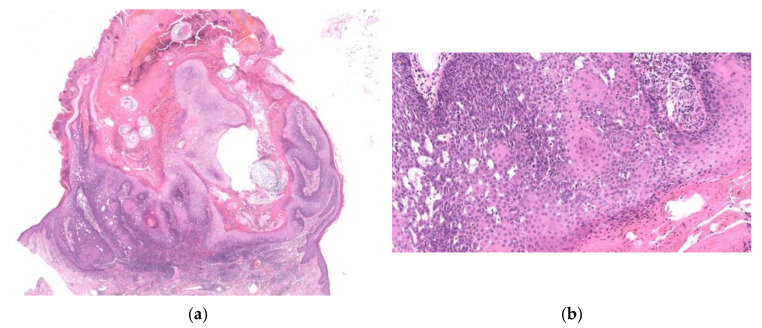
Histopathological findings of CSK. The lesion is crateriform with finger-like exophytic projections (**a**), showing hyperkeratosis and acanthosis with proliferation of basaloid cells (**b**). Pseudohorn cysts (**a**) and squamous eddies (**b**) are evident.

CSK is an exo-endophytic lesion, often having finger-like exophytic projections, which features hyperkeratosis and acanthosis with the proliferation of basaloid cells. Pseudohorn cysts are often evident (Figure 9).

### 4.2. Other Malignant Neoplasms

#### 4.2.1. Crateriform (Papillated) Bowen Disease

**Figure 10:**
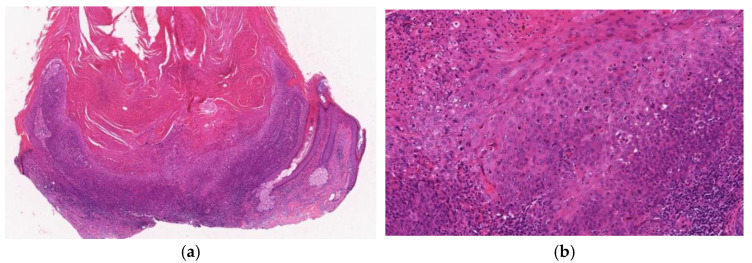
Histopathological findings of crateriform Bowen disease. The lesion shows crateriform and exo-endophytic proliferation with a central keratotic plug and overhanging epithelial lip-like structures (**a**). Typical features of Bowen’s disease, which are full-thickness dysplasia with markedly atypical keratinocytes, are seen in the epidermis (**b**).

Crateriform Bowen’s disease is an exo-endophytic lesion with a central keratotic horn [9,20]. It is formed from contiguous, keratinizing lobules and has overhanging epithelial lip-like structures (Figure 10). The typical features of Bowen’s disease (full-thickness dysplasia of the epidermis with markedly atypical keratinocytes, including multinucleated cells and dyskeratotic cells, with sparing of the basal cell layer) are observed at the sides of the epithelial lip-like structures and in the neoplastic lobules (Figure 10).

#### 4.2.2. Crateriform SCC Arising from Actinic Keratosis (cSCC)

**Figure 11:**
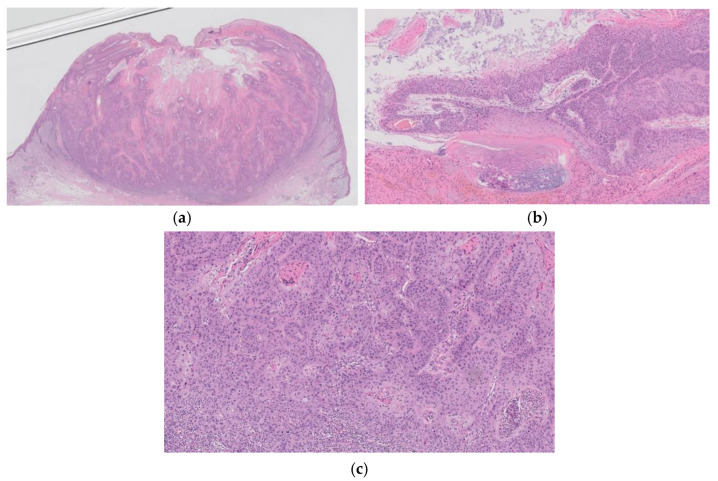
Histopathological findings of cSCC. A multilobular crateriform lesion (**a**) with epithelial lip-like structures (**b**) is observed. The full epidermal thickness of atypical keratinocytes with bowenoid features is evident at the base of the crater (**c**). There are no large, pale pink keratinizing cells with a glassy appearance featuring isthmic differentiation in the lobules (**c**). The histopathological features of solar keratosis are also observed in the periphery of the lesion (**b**).

This type of SCC is an exo-endophytic lesion exhibiting the full thickness of atypical keratinocytes with bowenoid features in the epidermis and into the dermis [9,15] (Figure 11). Epithelial lip-like structures may also be observed (Figure 11). There is no follicular (isthmic) differentiation, namely, no large pale pink keratinizing cells with a glassy appearance, in the lobules. A solar keratosis (bowenoid type) maybe be noted in the lesion or at its periphery (Figure 11).

#### 4.2.3. Crater Form of Infundibular SCC

**Figure 12:**
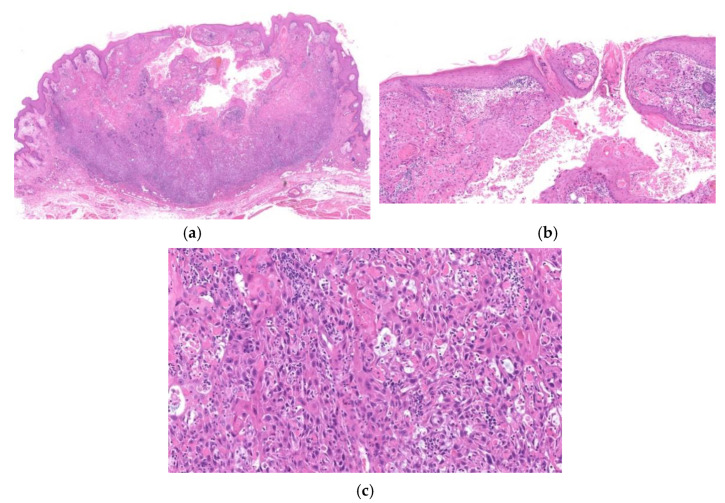
Histopathological findings of crater form of infundibular SCC. The lesion has a crateriform KA-like configuration with a central low keratin-filled ulcer (**a**). The tumor shows neoplastic aggregates of SCC expanding from a follicular infundibulum (**b**) and neoplastic cells invade deeply into the dermis (**a**,**c**). The features of KA or features of bowenoid dysplasia (solar keratosis or Bowen’s disease) are absent in the interfollicular epidermis (**a**).

The term follicular SCC was first proposed for folliculocentric SCC [21], and Kossard et al. [22] were the first to advocate the concept of SCC with infundibular differentiation as a subset of follicular carcinoma and introduced the descriptive term infundibulocystic SCC. Then, Misago et al. [20] focused on crater/ulcerated infundibular SCC, which was originally described as poorly differentiated infundibulocystic SCC by Kossard and colleagues [19]. The crater form of infundibular SCC has an exo-endophytic configuration with central ulceration or crusting and exhibits neoplastic aggregates of SCC expanding from a follicular infundibulum and neoplastic cells invade deeply into the dermis (Figure 12) [11]. On one or both sides of the lesion, epithelial lip-like structures may sometimes be noted in the periphery. Two or three contiguous follicular infundibula are involved along with proliferation of atypical keratinocytes. The neoplastic infundibular canal-like structures are composed of atypical bowenoid keratinocytes with parakeratosis, in contrast with the laminated keratinization of infundibular canals without nuclear atypia in KA. It is essential to confirm the absence of features of KA or features of bowenoid dysplasia (solar keratosis or Bowen’s disease) in the interfollicular epidermis (Figure 12) [11,23].

## 5. Data of Histopathological Diagnosis of Lesions Clinically Diagnosed as KA

Ansai, the co-author, reported the histopathological diagnosis of 1527 patients who were clinically diagnosed with KA at a Japanese institution [24]. Those lesions were most frequently located on the face (in approximately two-thirds). In 999 patients (65.4%), the histopathological architecture of KA was observed (KA lesion). The mean age at resection of the KA lesion (68.3 ± 15.1 years old) was significantly higher for these patients than for those without KA histopathological architecture (non-KA lesion) (61.0 ± 20.5 years old). In sun-exposed areas, the rate of KA lesions was high; 28.5% of the patients had malignant neoplasms, including SCC, especially patients over 60 years old, and 39.0% of cases were malignant. The rate of malignant lesions was higher in sun-exposed areas in elderly patients. The mean age at resection of malignant lesions (77.5 ± 11.5 years old) was significantly higher than that for benign lesions (61.1 ± 17.3 years old). The 1527 cases included 1397 (85.9%) epithelial tumors (including KA, verruca vulgaris, inverted follicular keratosis, trichofolliculoma and molluscum contagiosa) 99 (8.5%) non-epithelial tumors (including dermatofibroma, pyogenic granuloma, neurofibroma, xanthogranuloma, etc.), and 31 (2.0%) inflammatory lesions (including prurigo nodularis, etc.). Based on our impression, clinical differential diagnosis of crateriform epithelial tumors is very difficult. We consider that there is no certain clinical feature that differentiate benign crateriform tumors, especially solitary KA, from malignant ones, other than clinical course of the lesion, although CFV that is frequently observed benign crateriform tumor, shows long-standing course. Based on these findings, lesions clinically suspected as KA should be totally resected as soon as possible, especially on the faces of elderly patients.

## 6. Natural Course of KA and Related Lesions after Partial Biopsy

Takai and colleagues reported the clinical courses in 66 cases of KA and related lesions after partial biopsy [10]. They histopathologically classified these lesions into five types: (1) solitary KA at various stages (53 lesions); (2) KA-like SCC (3 lesions); (3) KA with malignant transformation (3 lesions); (4) infundibular SCC (5 lesions); and (5) crateriform SCC arising from solar keratosis (2 lesions). They analyzed the clinical course in each group. The regression rate of KA was 98.1% and that of KA-like SCC/KA with malignant transformation was 33.3%. No regression was observed in either infundibular SCC or crateriform SCC arising from solar keratosis. Thus, KA is a distinct entity that should be distinguished from other types of SCC with crateriform architecture based on the high frequency of regression. The regression rate of 33.3% in KA-like SCC/KA with malignant transformation indicated that KA lesions with a SCC component retain the potential for regression. However, this also suggested that KA is biologically unstable and some KA evolves into conventional SCC with a gradual loss of the capacity for the spontaneous regression. Infundibular SCC and crateriform SCC arising from solar keratosis are fundamentally different from KA, not only according to the histopathological findings, but also based on the biological properties. Thus, the classification we present in this article is reasonable in terms of the biological behavior of each neoplasm.

## 7. Incidence of Crateriform Epithelial Neoplasms

We previously reported the incidence of 380 epidermal crateriform tumors using our classification [12]. There were 214 cases of KA (56.3%), 76 cases of CFV (20%), 45 cases of KA with a conventional SCC component (11.8%), 12 cases of CSK and crateriform Bowen’s disease (3.2%), 11 cases of cSCC (2.9%) and 10 cases of infundibular SCC (2.6%). Benign crateriform neoplasms (CFV and CSK) and malignant crateriform neoplasms (KA with a conventional SCC component, Crateriform Bowen’s disease, cSCC and infundibular SCC) accounted for 88 lesions (23.3%) and 78 lesions (20.5%), respectively (Table 2). A total of 259 lesions at least partly had histopathological features of KA (KA and KA with a conventional SCC component), among which 45 (17.4%) had a SCC component. The incidence of SCC developing in KA was influenced by the patient’s age, being 8.3% in patients younger than 70 years old and increasing to 24.3% in those over 70. In this case, cSCC developed much more frequently in women than in men, CSK exhibited no sex difference, and the other lesions displayed a male predominance. The average age of the patients with malignant crateriform neoplasms was 70 years or older, whereas the average age of patients with benign crateriform neoplasms or KA was under 70 years. The average size of all types of lesions was approximately 1 cm. The mean duration of CFV was 14 months, which was the longest among the 7 types of neoplasms. Data for KA suggested that it progresses to the next stage every 2–3 months. The mean duration of infundibular SCC was 3.4 months, suggesting that it grows faster than the other malignant crateriform neoplasms. The sites of 366/380 lesions are summarized in Table 2. Most of the lesions developed on sun-exposed areas (head, face, neck, dorsum of hand and forearm). In particular, malignant crateriform neoplasms developed on sun-exposed areas (94.7%, 71/75). Of the 366 lesions, 232 (63.4%) were on the face, among which 138 (59.5%), 65 (28%) and 29 (12.5%) were KA, malignant crateriform neoplasms and benign crateriform neoplasms, respectively. All 10 infundibular SCCs developed on the face in elderly patients (mean age: 73 years, range: 59 to 87 years).

## 8. Conclusions

Complete surgical excision of the lesion is the most effective therapy for solitary KA. Therefore, we recommend complete excision of the lesion when KA is clinically suspected, especially when the lesion is located on a sun-exposed area in an elderly patient. If complete excision is impossible, partial excision of a sufficient specimen with intact architecture is required. In such a case, however, careful investigation after biopsy will be needed, even if the histopathological diagnosis is KA, because there is some possibility that a conventional SCC lesion remains in the residual tissue [25].

As mentioned above, solitary KA is a benign epithelial neoplasm with follicular differentiation that sometimes grows conventional SCC within it and is different from conventional SCC. We consider these to be the true characteristics of solitary KA.

## Figures and Tables

**Table 1:** Our classification of epithelial crateriform tumors.

Benign Neoplasms	Malignant Neoplasms
Crateriform verruca (CFV)	Crateriform (Papillated) Bowen disease
Crateriform seborrheic keratosis (CSK)	
	KA with conventional SCC component (KASCC)
Keratoacanthoma (KA)	Crateriform SCC arising from actinic keratosis (cSCC)
	Crater form of infundibular SCC

SCC: squamous cell carcinoma.

**Table 2:** Incidence of crateriform epithelial neoplasms.

Tumor		Case
CFV		76 (20.0%)
CSK		12 (3.2%)
KA	early/proliferative	85 (22.4%)
	well-developed	82 (21.6%)
	regressing/regressed	47 (12.4%)
	total	214 (56.3%)
Crateriform Bowen disease		12 (3.2%)
KAs with a conventional SCC		45 (11.8%)
cSCC		11 (2.9%)
Crateriform infundibular SCC		10 (2.6%)

## Data Availability

The data presented in this study are available in reference [10,12,24].
